# Poulticing

**Published:** 1907-08-17

**Authors:** Alexander Don

**Affiliations:** Park House, Dundee


### The General Practitioner's Column.

[Contributions to this Column are invited, and if accepted will be paid for.]

### POULTICING.

By ALEXANDER DON, M.B.C.M., F.R.C.S.Edin., Park House, Dundee.

In these days of serum-therapy, opsonins, high-frequency, and other methods of treatment, one may search widely in medical literature ere one comes across the word poultice; but those of us who plod our weary ways through suffering humanity, dealing with the common ills that flesh is heir to, often find the homely poultice of considerable service as a therapeutic agent. Time was—nay, is, with many of us—when the poultice was regularly applied in all inflammatory conditions, and no one would have thought of treating a bronchitis or a boil without a liberal supply of linseed meal. But so far has the pendulum swung to the other extreme, that poultices are excluded from our best hospitals and tabooed by both physicians and surgeons who wish to be considered up to date. The general practitioner who practises with success among the middle and lower classes finds in them a convenient method of attaining the same results as his fashionable brother gets by other methods; and I venture in this short article to face the sneers of these latter by saying something in favour of this handy remedy. First of all, it is an absolutely aseptic application, therefore surgically inoffensive. Applied to an unbroken boil or other acute inflammation it is extremely grateful to the sufferer; and, while it soothes the cutaneous nerves by relaxing the tissues, it also increases the blood supply, thus bringing to the affected area a greater supply of phagocytes. It thus hurries on the local action either towards repair or towards pus-formation and limits the destruction of tissue, and it can be renewed as necessary by the patient's friends without the superintendence of the doctor or other skilled assistance.

When the abscess bursts, or when it is nearing this stage, a piece of boracic lint or gauze wrung out of boiling water and placed below the poultice will absorb the discharge and prevent the formation of the crop of boils which is usually seen when the poultice is kept applied next the skin. And if the abscess does not burst, then the physician can assist the process with his lancet when he thinks the abscess sufficiently circumscribed. Thus he attains the same object as does the surgeon with his Bier's cupping apparatus, or the physician with his anti-serums or vaccines—namely, phagocytosis. As a remedy for internal pain, such as pleurisy or peritonitis, no substitute of equal value both for the patient and the physician has been proposed. From the physician's point of view it tides over the time when otherwise he would have to give morphia, and the patient waits patiently, believing that everything possible is being done for him; while the friends are pleased to think that they are the real ministering angels. At a subsequent visit the doctor is then able fully to appreciate the condition of his patient, which is not an easy matter when opium has been administered.

As an antispasmodic in asthma and in the first stages of acute bronchitis and pneumonia there is so far no superior remedy, for fomentations are certainly not so aseptic, and they are less comforting to the patient, have to be applied oftener, wet the bed and body clothes, and do not satisfy the friends so well as the old-fashioned poultice. How often does one find, on making a second visit, that the patient says he was so bad that he had to be poulticed? and we cannot deny that he and his relatives are pleased with the good they have effected.

Why, then, have poultices been so unanimously rejected by the foremost in our profession? I believe that it was the abuse of poulticing, as of blood-letting, that ousted this method of treatment from modern text-books. Some practitioners even yet never see a bronchitis without advising poulticing, whether the secretions be loose or sticky, the patient young or old or robust; and there is no doubt that so applied they do an infinite amount of harm. And so, too, we are told does the serum-treatment if the injections are made during the negative phase. We have no equivalent index like the opsonic to tell us when to stop or when to apply a poultice if we have not a well-balanced judgment to guide us; and this is as difficult to acquire as an opsonic index is in point of cost beyond the reach of a general practitioner's patients.

In surgery, again, the continued application of poultices to an open wound not only does no good, but infinite harm in closing up the secretions and converting an open sore into an absorbing cavity, while the organisms in the secretions that escape are afforded a warm cultivating medium in which they rapidly develop and infect the surrounding healthy tissues.

In surgery the advantages of poulticing are:—It increases local hyperæmia and phagocytosis; acts as a moist absorbent dressing; and relieves the general practitioner of the somewhat constant dressing required in some surgical cases where the patient cannot well afford the cost of his attendance, while it satisfies the patient and friends by giving them constant employment.

In medicine the indications for use are:—(1) To relieve pain in all inflammatory conditions; (2) to increase the blood supply, and thus to facilitate repair: (3) to soothe nerve spasm, as in asthma, and to relieve the burning sensation of commencing congestion, etc., as in bronchitis and pneumonia.

Routine poulticing in chest troubles, especially in children and the aged; poulticing through all the stages of every bronchitis and chest affection; and poulticing in abdominal pain for any length of time without a definite diagnosis—*e.g.*, in peritonitis due to appendicitis or malignant disease—should be avoided.

